# Reliability of computerized image analysis for the evaluation of serial synovial biopsies in randomized controlled trials in rheumatoid arthritis

**DOI:** 10.1186/ar1757

**Published:** 2005-05-12

**Authors:** Jasper J Haringman, Marjolein Vinkenoog, Danielle M Gerlag, Tom JM Smeets, Aeilko H Zwinderman, Paul P Tak

**Affiliations:** 1 Division of Clinical Immunology and Rheumatology, Academic Medical Center/University of Amsterdam, The Netherlands; 2 Department of Clinical Epidemiology and Biostatistics, Academic Medical Center/University of Amsterdam, The Netherlands

## Abstract

Analysis of biomarkers in synovial tissue is increasingly used in the evaluation of new targeted therapies for patients with rheumatoid arthritis (RA). This study determined the intrarater and inter-rater reliability of digital image analysis (DIA) of synovial biopsies from RA patients participating in clinical trials. Arthroscopic synovial biopsies were obtained before and after treatment from 19 RA patients participating in a randomized controlled trial with prednisolone. Immunohistochemistry was used to detect CD3^+ ^T cells, CD38^+ ^plasma cells and CD68^+ ^macrophages. The mean change in positive cells per square millimetre for each marker was determined by different operators and at different times using DIA. Nonparametric tests were used to determine differences between observers and assessments, and to determine changes after treatment. The intraclass correlations (ICCs) were calculated to determine the intrarater and inter-rater reliability. Intrarater ICCs showed good reliability for measuring changes in T lymphocytes (R = 0.87), plasma cells (R = 0.62) and macrophages (R = 0.73). Analysis by Bland–Altman plots showed no systemic differences between measurements. The smallest detectable changes were calculated and their discriminatory power revealed good response in the prednisolone group compared with the placebo group. Similarly, inter-rater ICCs also revealed good reliability for measuring T lymphocytes (R = 0.68), plasma cells (R = 0.69) and macrophages (R = 0.72). All measurements identified the same cell types as changing significantly in the treated patients compared with the placebo group. The measurement of change in total positive cell numbers in synovial tissue can be determined reproducibly for various cell types by DIA in RA clinical trials.

## Introduction

Rheumatoid arthritis (RA) is characterized by chronic and symmetric inflammation of synovial joints [1,2]. Although the aetiology of RA is still unknown, it is thought of as an autoimmune disease with the synovial tissue (ST) being its primary target. The microscopic appearance of RA ST includes marked intimal lining layer hyperplasia due to increased numbers of fiboblast-like synoviocytes and intimal macrophages, and accumulation of macrophages, T cells, B cells, plasma cells, dendritic cells, mast cells, natural killer cells and neutrophils in the synovial sublining layer [3]. Developments in synovial biopsy techniques, especially arthroscopy, have resulted in easier access to human ST. It is now possible to select ST from many sites within large and small joints, even in the earliest phases of disease, enhancing studies of aetiology, prognosis and response to treatment [4].

Analysis of biomarkers in ST is increasingly being used in the evaluation of new targeted therapies in RA patients [5]. Numerous studies have suggested consistent associations between rapidity and magnitude of both clinical and immunohistological responses. It was shown that, especially within the ST, the number of infiltrating sublining macrophages can be used as a biomarker of clinical efficacy in relatively small studies of short duration [6,7]. Therefore, change in synovial sublining macrophages may be used as a biomarker for the evaluation of novel antirheumatic therapies. In addition to screening for possible efficacy, this approach provides insight into the mechanism of action of treatment.

Within this setting, reliable and validated methods for studying the ST are pivotal. The use of computerized or digital image analysis (DIA) has greatly facilitated the evaluation of ST. The major advantage of DIA is standardization of image acquisition and processing, minimizing variance, and the ability to quantify the actual stained area together with staining intensity in a time efficient manner [8,9]. This allows analysis of large numbers of stained sections. Strong correlations were observed between CIA, semiquantitative scoring and manual counting for analysis of ST cellular markers, cytokines and adhesion molecules [10,11]. Although the reproducibility of measuring cytokine and cell adhesion molecule staining by DIA was reported to be within 10% [8], no formal studies investigating intrarater and inter-rater variability have yet been reported. Therefore, we designed a study to determine the intrarater and inter-rater reliability of this approach for the analysis of synovial biopsies from RA patients participating in clinical trials.

## Materials and methods

### Patients and samples

Arthroscopic synovial biopsies were obtained before and 2 weeks after treatment in 19 patients who participated in a double-blind, placebo-controlled, single-centre study with prednisolone, as reported earlier [6]. All patients included had RA according to the 1987 criteria proposed by the American College of Rheumatology [12] and were on a stable regimen of disease-modifying antirheumatic drugs (methotrexate, sulphasalazine, hydroxychloroquine or leflunomide, or a combination of these) for at least 28 days before inclusion in the study. Ten out of the 19 patients received prednisolone and nine received placebo treatment. Needle arthroscopy of an actively inflamed joint (knee, ankle, or wrist) was performed under local anaesthesia in all patients before treatment and in the same joint after treatment. The procedures for needle arthroscopy were performed as described previously in detail [13,14]. During each procedure, biopsies were taken from six or more sites throughout the joint to minimize sampling error [15,16]. These specimens were directly collected *en bloc *in a mold embedded in Tissue Tek OCT (Miles diagnostics, Elkhart, IN, USA) and subsequently snap frozen by immersion in methylbutane (-80°C). The frozen blocs were stored in liquid nitrogen until they were processed. The study was approved by the Medical Ethics Committee of the Academic Medical Center, Amsterdam, The Netherlands, and all patients provided informed consent before start of the study.

### Immunohistochemical analysis

From each tissue sample, consisting of six different biopsy samples, serial sections were cut with a cryostat (5 μm) and stained with the following antibodies to analyze the major cell populations in the synovium: anti-CD68 (EMB11; Dako, Glostrup, Denmark), anti-CD38 (HB-7; Becton Dickinson) and anti-CD3 (SK7; Becton Dickinson, Erembodegem, Belgium). Sections with nonassessable tissue, defined as the absence of an intimal lining layer, were not analyzed. For control sections, the primary antibodies were omitted or irrelevant antibodies were applied. Staining for cellular markers was performed using a three-step immunoperoxidase method, as was previously described [17].

### Digital image analysis

**Figure 1:**
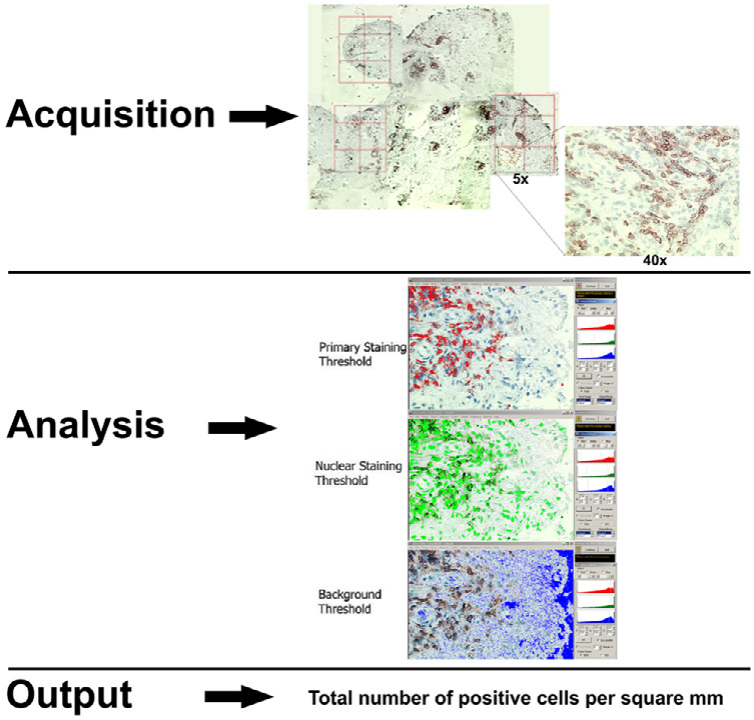
Acquisition, analysis and output for a digital image analysis system. Acquisition and analysis of immunohistochemical staining of CD3^+ ^T lymphocytes in synovial tissue using a digital image analysis system [10]. Three different areas from each biopsy sample, which are representative of the whole tissue section are selected. During analysis, staining thresholds are set for primary staining (i.e. CD3^+ ^T lymphocytes), nuclear staining and background staining. The output is generated in a spreadsheet as the total number of positive cells per square millimetre of synovial tissue.

After immunohistochemical staining, all coded sections were randomly analyzed by computer-assisted image analysis (Fig. 1). For all markers, 18 high-power fields were analyzed. The images of the high-power fields were analyzed using the Qwin analysis system (Leica, Cambridge, UK), as described previously in detail [10,11].

For determination of intrarater reliability, one observer performed the acquisition and analysis twice with an interval of 4 weeks in between (OB1 t0 and OB1 t1, respectively). To determine the inter-rater reliability, acquisition of images and analysis were performed independently by two other experienced observers (OB2 and OB3). All observers were blinded regarding clinical data. For each measurement all observers independently set their own threshold levels regarding the detection of stained antigen, nuclear staining and background staining. After the analysis, all observers independently calculated the mean change in the total number of positive cells per square millimetre of ST for each marker.

### Statistical analysis

The nonparametric Friedman test and the Wilcoxon signed rank test were used to identify differences in the detection of the change in positive cell numbers per marker in the whole patient group, between observers and between assessments. The intrarater and inter-rater reliability was quantified by means of the intraclass correlation coefficient (ICC) of agreement [18]. In addition, scatter plots, in accordance with methods reported by Bland and Altman [19], were constructed to show differences in the change in positive cells between two measurements from one observer. The smallest detectable changes (SDCs), representing the smallest change in scores that can be deemed to be a 'real' change [20], for the intra-observer variances was calculated and used to evaluate their disciminatory power. The nonparametric Mann–Whitney U-test was used to determine whether each analysis detected differences in the change of positive cell numbers when the placebo group was compared with the prednisolone-treated group.

## Results

### Intrarater reliability

The mean numbers of CD3^+ ^T lymphocytes, CD38^+ ^plasma cells and CD68^+ ^sublining macrophages before and after intervention for two analyses by the same observer at different time points (OB1 t0 and OB1 t1) are shown in Table 1. There were no significant differences in the mean change in T cells, plasma cells and macrophages in the total population between the two measurements.

**Figure 2:**
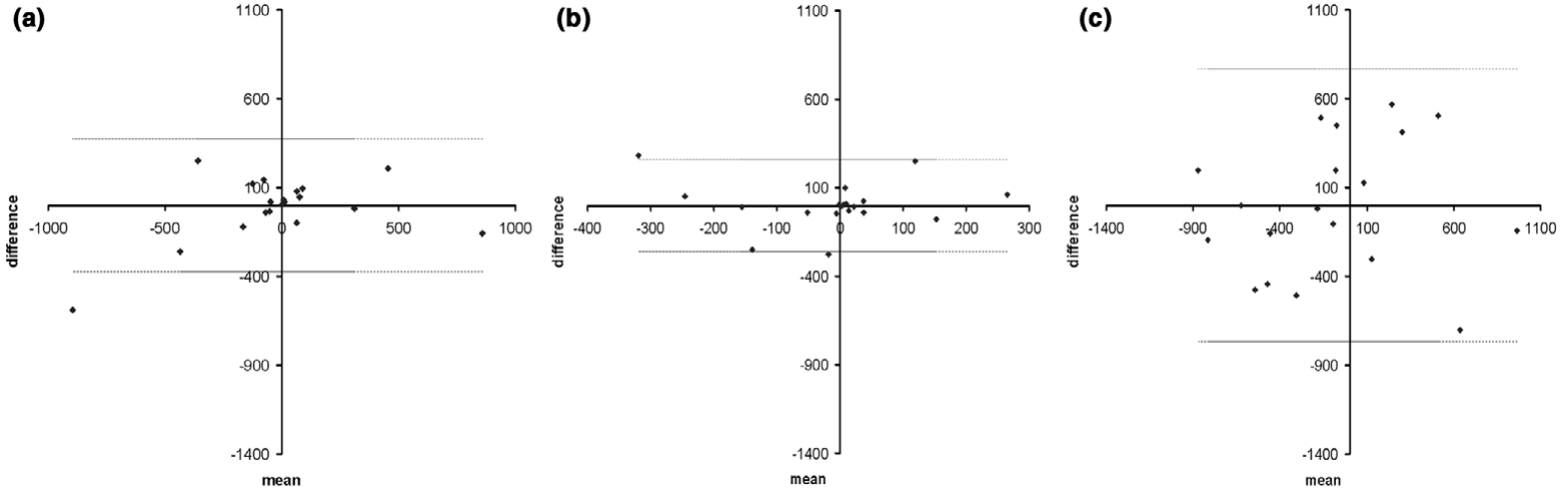
Mean change in number of positive cells versus the difference in change in positive cells. Shown are scatter plots of the mean change in the number of positive cells versus the difference in change of positive cells between two measurements by observer 1 for **(a) **CD3^+ ^lymphocytes, **(b) **CD38^+ ^plasma cells and **(c) **CD68^+ ^macrophages. The dotted line represents the mean ± 2 × standard deviation.

The overall correlations between the first and second analysis by the same observer were good. For the measurement of the change in CD3^+ ^T lymphocytes, CD38^+ ^plasma cells and CD68^+ ^macrophages, the single rater and average of rater ICCs were calculated and are shown in Table 2. The relations between the two measurements by the single observer are plotted in Fig. 2. There were no systemic differences between the two measurements for each marker, but the variation was rather large. An analysis of the between patient variances and within patient variances is provided in Table 2.

The SDC, averaged for the number of readings, for CD3^+ ^lymphocytes was 182, for CD38^+ ^plasma cells it was 128, and for CD68^+ ^macrophages it was 306. When these estimates were used to identify those patients who responded to the treatment (i.e. had a reduction in positive cell numbers exceeding the SDC), for CD3^+ ^lymphocytes four of the 10 patients in the prednisolone group responded versus none of the nine patients in the placebo group; for CD38^+ ^plasma cells four of the 10 patients in the prednisolone group responded versus one of the nine patients in the placebo group; and for CD68^+ ^macrophages seven out of the 10 patients in the prednisolone group responded versus none of the nine patients in the placebo group.

To determine whether the same observer identified the same differences in the synovial infiltrate after treatment at different time points, we determined whether there were significant differences in the change in T cells, plasma cells and macrophages between the placebo group and the prednisolone-treated group for each measurement. At both time points there was, on average, a significant reduction in the number of CD3^+ ^lymphocytes and CD68^+ ^macrophages in the prednisolone-treated patients as compared with placebo (Table 1), whereas on average there were no significant changes in the number of CD38^+ ^plasma cells.

### Interrater reliability

The mean number of T cells, plasma cells and macrophages before and after intervention measured by the other two observers (OB2 and OB3) are also shown in Table 1. There were no statistically significant differences in the mean change in positive cells between the analyses by the three observers (OB1 t0, OB2 and OB3).

When the overall correlations between the analyses of the three observers were calculated the ICCs (single and average of raters) appeared to be good for CD3^+ ^lymphocytes, CD38^+ ^plamsa cells and CD68^+ ^macrophages (Table 2). An analysis of between patient variances and the within patient variances is also provided in Table 2.

To determine whether all three observers identified the same differences in the synovial infiltrate after treatment, we determined whether there were significant differences in the change in T cells, plasma cells and macrophages between the placebo group and the prednisolone-treated group for each measurement. The measurements by all three observers showed, on average, a significant reduction in the number of CD3^+ ^lymphocytes and CD68^+ ^macrophages in the prednisolone-treated patients versus placebo (Table 1), whereas, on average, there were no significant changes in the number of CD38^+ ^plasma cells.

## Discussion

This study investigated the intra- and interobserver reliability of assessment of the change in ST T cells, plasma cells, and macrophages quantified by DIA. Tissue samples were obtained from RA patients participating in a single-centre, placebo-controlled clinical trial with prednisolone. There were no significant differences in measurement of the mean change in T cells, plasma cells and macrophages between the three observers, or for different measurements by one observer. ICCs revealed good agreement between measurements. All observers and all measurements identified, on average, significant reductions in T cells and macrophages but not in plasma cells in the prednisolone group compared with placebo.

It can be anticipated that there will be an upsurge in randomized controlled trials investigating novel biological agents and small molecules in terms of their safety and efficacy. Thus, sensitive, validated and reliable measurements to screen for potential efficacy in an early phase of drug development are clearly needed. Clinical outcome measures have historically been used as primary end-points, but their reliability may be limited in small proof-of-principle studies. For clinical measurements such as the tender and swollen joint count, ICCs have been reported to vary between 0.15 and 0.85 for inter-rater variability and between 0.67 and 0.95 for intrarater variability [21]. Radiographic measurements, with the use of conventional X-ray films, show good reliability in most studies but they are not useful in short-term clinical trials [21]. The use of magnetic resonance images is promising, with acceptable inter-rater ICC for global synovitis scores and bone erosions, although optimal scoring systems are yet to be developed [22].

In light of the need to screen various compounds for potential efficacy in small numbers of patients and because of recent technical developments, we believe that our thinking about clinical trials is about to change dramatically. Clinical studies conducted during early phases of drug development will increasingly consist of small trials with a high density of biological data [23]. Consistent with this notion, serial ST analysis with evaluation of biomarkers was recently included in several randomized clinical trials of both disease-modifying anti-rheumatic drugs and biological agents [6,13,24-27]. These and other studies showed consistent relationships between the magnitude of synovial changes and clinical response. In particular, the change in infiltrating sublining macrophages was identified to be a potent and sensitive synovial biomarker [6,7].

ST can easily and safely be obtained as a result of the introduction of small-bore arthroscopes and the development of local and regional anaesthesia protocols. Despite heterogeneity in the ST within a single joint, it has been shown that representative measures of synovial inflammation can be obtained by examining a limited area of tissue [15,28,29]. Previous work [10,11] has also shown that DIA is a sensitive, time efficient method for quantifying both the number of stained cells and the staining intensity, with good correlations with both manual counting and semiquantative scoring.

Although DIA is described as reliable and objective, little is known about the variability and reliability of this tool. Variation in measurements may result from a limited number of factors with this approach. In our system the observer selects three different areas of each six high-power fields from one slide, which is composed of six biopsy samples from six different sites in the joint. This is done in such a way that a representative area is selected, and this requires extensive training and experience with the histopathological morphology of ST. After scanning the representative high-power fields, the images are analyzed by setting threshold values for the stained antigen, nuclear staining and background staining [10]. These thresholds are kept constant for all measurements with the same marker within a study, but could theoretically give rise to variation when set by different observers or by one observer at different times. In the present study it was shown that these variables did not result in different outcomes. There were good ICCs when the findings of three experienced observers or the findings of the same observer at different times were compared. Analysis by Bland–Altman plots showed no systemic differences with regard to the intra-observer measurements, and the SDCs showed good discriminatory power when applied to the treatment groups. In addition, all observers and all measurements identified the same cell types (T cells and macrophages) as decreasing significantly in the active treatment group compared with placebo. All measurements also identified a consistent trend toward reduced plasma cell numbers after corticosteroid treatment, which did not reach statistical significance, possibly because of the relative small number of patients included. Although this method does exhibit good agreement in detecting changes in histological markers, this does not necessarily mean that these results can be extrapolated to the expression of a given marker at a given time point, as used in cross-sectional studies of ST. In addition, it remains to be seen whether the same reliability holds true for determination of changes in secreted proteins, such as cytokines and chemokines.

## Conclusion

In conclusion, the findings of the present study show the reliability of ST analysis using a DIA system for the evaluation of serial synovial biopsy samples before and after treatment. This approach may be used for efficient quantification of synovial biomarkers in small proof-of-principle clinical trials.

## Competing Interests

The author(s) declare that they have no competing interests.

## Abbreviations

DIA = digital image analysis; ICC = intraclass correlation coefficient; RA = rheumatoid arthritis; SDC = smallest detectable change; ST = synovial tissue.

## Authors' contributions

JJH contributed to experiments, was responsible for data analysis and interpretation, and wrote the manuscript. MV and TJMS were responsible for both the set-up and performance of the experiments. DMG was responsible for including patients and collecting materials and data. AHZ coordinated and assisted in the statistical analysis of the data. PPT was responsible for planning the work and contributed to data analysis, interpretation and write up.

## Figures and Tables

**Table 1:** Numbers of positive cells before and after intervention

Cell type	Treatment	Observer 1 t0			Observer 1 t1			Intra-observer comparison	Observer 2			Observer 3			Inter- observer comparison
		
		Placebo	Prednisolone	*P*^a^	Placebo	Prednisolone	*P*^a^	*P*^b^	Placebo	Prednisolone	*P*^a^	Placebo	Prednisolone	*P*^a^	P^c^
CD3^+ ^T lymphocytes	Before	192 ± 246	358 ± 413	<0.05	110 ± 140	227 ± 225	<0.05	NS	179 ± 185	285 ± 250	<0.05	89 ± 110	112 ± 98	<0.05	NS
	After	387 ± 391	140 ± 150		299 ± 356	44 ± 62			470 ± 618	101 ± 94		196 ± 224	54 ± 66		
CD38^+ ^plasma cells	Before	56 ± 87	99 ± 130	NS	73 ± 134	116 ± 166	NS	NS	246 ± 307	397 ± 498	NS	145 ± 151	309 ± 380	NS	NS
	After	96 ± 127	37 ± 57		119 ± 149	42 ± 78			335 ± 411	132 ± 187		315 ± 416	83 ± 121		
CD68^+ ^macrophages	Before	804 ± 422	973 ± 419	<0.03	441 ± 422	572 ± 404	<0.03	NS	937 ± 292	1151 ± 254	<0.03	621 ± 445	724 ± 360	<0.03	NS
	After	972 ± 151	553 ± 342		632 ± 686	222 ± 278			984 ± 354	796 ± 306		720 ± 527	313 ± 291		

Shown are the mean numbers (± standard deviation) of CD3^+ ^T lymphocytes, CD38^+ ^plasma cells and CD68^+ ^sublining macrophages per square millimetre of synovial tissue before and after intervention, measured by one observer at two different time points (OB1 t0 and OB1 t1) and by two other observers (OB2 and OB3) for placebo-treated patients and prednisolone-treated patients. ^a^Nonparametric, unpaired, Mann–Whitney U-test for the comparison between placebo and prednisolone treatment. ^b^Nonparametric, paired, Wilcoxon signed rank test, for the comparison between OB1 t0 and OB1 t1 (intra-observer comparison). ^c^Nonparametric, paired, Friedman test, for the comparsion between the three observers (OB1 t0, OB2 and OB3).

**Table 2:** Estimates of the variance components (between and within patients) and of the intraclass correlations (single rater and average of raters)

Cell type	Intra-observer				Inter-observer			
	
	Between patients	Within patients	ICC	ICC of the mean of two observations	Between patients	Within patients	ICC	ICC of the mean of three observations
CD3^+ ^cells	11.59	1.73	0.87	0.93	10.13	4.85	0.68	0.86
CD38^+ ^cells	1.35	0.82	0.62	0.77	8.65	3.83	0.69	0.87
CD68^+ ^cells	20.32	7.35	0.73	0.85	18.92	7.19	0.72	0.89

ICC, intraclass correlation coefficient.

## References

[B1] O'Dell JR (2004). Therapeutic strategies for rheumatoid arthritis. N Engl J Med.

[B2] Firestein GS (2003). Evolving concepts of rheumatoid arthritis. Nature.

[B3] Tak PP, Bresnihan B (2000). The pathogenesis and prevention of joint damage in rheumatoid arthritis: advances from synovial biopsy and tissue analysis. Arthritis Rheum.

[B4] Bresnihan B, Tak PP (1999). Synovial tissue analysis in rheumatoid arthritis. Baillieres Best Pract Res Clin Rheumatol.

[B5] Tak PP (2000). Lessons learnt from the synovial tissue response to anti-rheumatic treatment. Rheumatology (Oxford).

[B6] Gerlag DM, Haringman JJ, Smeets TJ, Zwinderman AH, Kraan MC, Laud PJ, Morgan S, Nash AF, Tak PP (2004). Effects of oral prednisolone on biomarkers in synovial tissue and clinical improvement in rheumatoid arthritis. Arthritis Rheum.

[B7] Haringman JJ, Gerlag DM, Zwinderman AH, Smeets TJ, Kraan MC, Baeten D, McInnes IB, Bresnihan B, Tak PP (2005). Synovial tissue macrophages: highly sensitive biomarkers for response to treatment in rheumatoid arthritis patients. Ann Rheum Dis.

[B8] Youssef PP, Triantafillou S, Parker A, Coleman M, Roberts-Thomson PJ, Ahern MJ, Smith MD (1997). Variability in cytokine and cell adhesion molecule staining in arthroscopic synovial biopsies: quantification using color video image analysis. J Rheumatol.

[B9] Norazmi MN, Hohmann AW, Jarvis LR, Skinner JM, Stoll P, Bradley J (1990). The use of computer-assisted video image analysis in the enumeration of immuno-stained cells in tissue sections. J Immunol Methods.

[B10] Kraan MC, Haringman JJ, Ahern MJ, Breedveld FC, Smith MD, Tak PP (2000). Quantification of the cell infiltrate in synovial tissue by digital image analysis. Rheumatology (Oxford).

[B11] Kraan MC, Smith MD, Weedon H, Ahern MJ, Breedveld FC, Tak PP (2001). Measurement of cytokine and adhesion molecule expression in synovial tissue by digital image analysis. Ann Rheum Dis.

[B12] Arnett FC, Edworthy SM, Bloch DA, McShane DJ, Fries JF, Cooper NS, Healey LA, Kaplan SR, Liang MH, Luthra HS (1988). The American Rheumatism Association 1987 revised criteria for the classification of rheumatoid arthritis. Arthritis Rheum.

[B13] Kraan MC, Reece RJ, Barg EC, Smeets TJ, Farnell J, Rosenburg R, Veale DJ, Breedveld FC, Emery P, Tak PP (2000). Modulation of inflammation and metalloproteinase expression in synovial tissue by leflunomide and methotrexate in patients with active rheumatoid arthritis. Findings in a prospective, randomized, double-blind, parallel-design clinical trial in thirty-nine patients at two centers. Arthritis Rheum.

[B14] Kraan MC, Reece RJ, Smeets TJ, Veale DJ, Emery P, Tak PP (2002). Comparison of synovial tissues from the knee joints and the small joints of rheumatoid arthritis patients: Implications for pathogenesis and evaluation of treatment. Arthritis Rheum.

[B15] Dolhain RJ, Ter Haar NT, De Kuiper R, Nieuwenhuis IG, Zwinderman AH, Breedveld FC, Miltenburg AM (1998). Distribution of T cells and signs of T-cell activation in the rheumatoid joint: implications for semiquantitative comparative histology. Br J Rheumatol.

[B16] Boyle DL, Rosengren S, Bugbee W, Kavanaugh A, Firestein GS (2003). Quantitative biomarker analysis of synovial gene expression by real-time PCR. Arthritis Res Ther.

[B17] Tak PP, van der Lubbe PA, Cauli A, Daha MR, Smeets TJ, Kluin PM, Meinders AE, Yanni G, Panayi GS, Breedveld FC (1995). Reduction of synovial inflammation after anti-CD4 monoclonal antibody treatment in early rheumatoid arthritis. Arthritis Rheum.

[B18] Deyo RA, Diehr P, Patrick DL (1991). Reproducibility and responsiveness of health status measures. Statistics and strategies for evaluation. Control Clin Trials.

[B19] Bland JM, Altman DG (1986). Statistical methods for assessing agreement between two methods of clinical measurement. Lancet.

[B20] Bruynesteyn K, Boers M, Kostense P, van der LS, van der HD (2005). Deciding on progression of joint damage in paired films of individual patients: smallest detectable difference or change. Ann Rheum Dis.

[B21] Lassere MN, van der HD, Johnson KR, Boers M, Edmonds J (2001). Reliability of measures of disease activity and disease damage in rheumatoid arthritis: implications for smallest detectable difference, minimal clinically important difference, and analysis of treatment effects in randomized controlled trials. J Rheumatol.

[B22] Lassere M, McQueen F, Ostergaard M, Conaghan P, Shnier R, Peterfy C, Klarlund M, Bird P, O'Connor P, Stewart N (2003). OMERACT Rheumatoid Arthritis Magnetic Resonance Imaging Studies. Exercise 3: an international multicenter reliability study using the RA-MRI Score. J Rheumatol.

[B23] Liu ET, Karuturi KR (2004). Microarrays and clinical investigations. N Engl J Med.

[B24] Cunnane G, Madigan A, Murphy E, Fitzgerald O, Bresnihan B (2001). The effects of treatment with interleukin-1 receptor antagonist on the inflamed synovial membrane in rheumatoid arthritis. Rheumatology (Oxford).

[B25] Smeets TJ, Kraan MC, van Loon ME, Tak PP (2003). Tumor necrosis factor alpha blockade reduces the synovial cell infiltrate early after initiation of treatment, but apparently not by induction of apoptosis in synovial tissue. Arthritis Rheum.

[B26] Haringman JJ, Kraan MC, Smeets TJ, Zwinderman KH, Tak PP (2003). Chemokine blockade and chronic inflammatory disease: proof of concept in patients with rheumatoid arthritis. Ann Rheum Dis.

[B27] Katrib A, Smith MD, Ahern MJ, Slavotinek J, Stafford L, Cuello C, Bertouch JV, McNeil HP, Youssef PP (2003). Reduced chemokine and matrix metalloproteinase expression in patients with rheumatoid arthritis achieving remission. J Rheumatol.

[B28] Rooney M, Condell D, Quinlan W, Daly L, Whelan A, Feighery C, Bresnihan B (1988). Analysis of the histologic variation of synovitis in rheumatoid arthritis. Arthritis Rheum.

[B29] Bresnihan B, Cunnane G, Youssef P, Yanni G, Fitzgerald O, Mulherin D (1998). Microscopic measurement of synovial membrane inflammation in rheumatoid arthritis: proposals for the evaluation of tissue samples by quantitative analysis. Br J Rheumatol.

